# On Teething of Infants

**Published:** 1865-10

**Authors:** 


### ON TEETHING OF INFANTS—ITS PREVALENT ERRORS, NEGLECTS AND DANGERS.

Their influence on the health, and as causes of death of children; including the dangers of teething powders, soothing powders, soothing syrups, etc., etc. Illustrated by cases.

BY HENRY HANKS.

This is a little work we take great pleasure in recommending to our professional brethren, one fraught with many valuable suggestions upon this very important subject.

This is a matter to which the attention of dentists should be directed far more than it has been. They have been disposed to overlook it, regarding it as more immediately within the sphere of the general physician; and the physicians too have been disposed to pass it by as a small matter, and thus far, as a general thing, its thorough investigation, and thorough and efficient treatment, have been neglected.

We have no controversy with the general physician, but the subject of dentition does come within the sphere of the dentist, and this work introduces the subject to us. It is well worthy of close reading, and though not elaborate, presents many valuable points in a very forcible manner. Let every dentist read it.

